# Mitochondrial DNA Haplogroups Influence Oxidative Stress Profiles and Susceptibility to Metabolic Syndrome in an Asian Population

**DOI:** 10.3390/antiox15091155

**Published:** 2026-09-11

**Authors:** Shao-Wen Weng, Yu-Han Lin, Shih-Hsuan Chen, Tsu-Kung Lin, Pei-Wen Wang, Chia-Wei Liou

**Affiliations:** 1 Department of Metabolism, Kaohsiung Chang Gung Memorial Hospital and Chang Gung University College of Medicine, Kaohsiung 83301, Taiwan; wengsw99@adm.cogm.org.tw (S.-W.W.); wangpw@adm.cgmh.org.tw (P.-W.W.); 2 Center for Mitochondrial Research and Medicine, Kaohsiung Chang Gung Memorial Hospital and Chang Gung University College of Medicine, Kaohsiung 83301, Taiwan; cgmhlinyh202@cgmh.org.tw (Y.-H.L.); tklin@adm.cgmh.org.tw (T.-K.L.); 3 Department of Neurology, Kaohsiung Chang Gung Memorial Hospital and Chang Gung University College of Medicine, Kaohsiung 83301, Taiwan; b9502054@adm.cgmh.org.tw

**Keywords:** mitochondrial DNA haplogroup, oxidative stress, metabolic syndrome, thiobarbituric acid-reactive substances (TBARS), thiols, mitochondrial genetics

## Abstract

Mitochondrial DNA (mtDNA) haplogroups influence mitochondrial function and reactive oxygen species (ROS) production, potentially modulating susceptibility to metabolic disorders. This study investigated the associations between mtDNA haplogroups, systemic oxidative stress, and metabolic syndrome in 2486 Taiwanese individuals. Serum thiobarbituric acid-reactive substances (TBARS) and free thiols were measured as markers of oxidative and antioxidative status, respectively, while mtDNA haplogroups were determined using multiplex PCR and Luminex genotyping. Macrohaplogroups N (51%) and M (49%) were predominant, with haplogroups B, F, D, and M7 most frequently observed. Although the overall haplogroup distribution is genetically similar to populations in Southern China, the distribution among the Taiwanese population shows distinct differences. Haplogroup B was significantly associated with higher TBARS levels, lower thiol concentrations, and an increased prevalence of diabetes and metabolic syndrome (OR 1.34, *p* = 0.004). Individuals with metabolic syndrome exhibited higher oxidative stress and lower antioxidative biomarker levels than those without the syndrome, while a progressive imbalance in oxidative–antioxidative status was observed with increasing numbers of metabolic syndrome components. These findings suggest that mtDNA haplogroups, particularly haplogroup B, may contribute to metabolic syndrome susceptibility through modulation of systemic oxidative stress.

## 1. Introduction

Oxidative stress generated by cells is known to be involved in the development of many chronic diseases in humans [1]. Mitochondria, as the cellular powerhouse, use oxygen for energy production; however, leakage of reactive oxygen species during this process is unavoidable [2]. Although cells possess antioxidant defense systems to neutralize these reactive species, an imbalance between their production and elimination leads to oxidative stress [3,4]. Thus, mitochondria are considered a major contributor to oxidative stress, and previous studies have suggested that this process may be closely related to mitochondrial DNA (mtDNA) variation [5]. Cellular model investigations have revealed that different mtDNA haplogroups, defined by specific combinations of mtDNA variants, exhibit distinct functional characteristics, including differences in ATP production efficiency and reactive oxygen species generation [6,7,8]. These mtDNA variations are distributed in varying proportions among different population groups worldwide [9]. Accordingly, certain mtDNA haplogroups may contribute to cellular vulnerability under conditions of oxidative stress, leading to population-specific susceptibility to various diseases.

Mitochondrial genetic variation is increasingly recognized as an important determinant of disease susceptibility and progression. In our previous studies, we demonstrated that, within Taiwanese populations of Chinese ancestry, specific mtDNA haplogroups are significantly associated with susceptibility to several complex diseases, including diabetes mellitus, Parkinson’s disease, and stroke [10,11,12]. More recently studies have further supported associations between mtDNA variation, mtDNA copy number, and susceptibility to diabetes and metabolic syndrome [13,14,15]. Notably, both disease onset and progression are closely linked to alterations in systemic oxidative stress, as reflected by circulating biomarkers such as thiobarbituric acid-reactive substances (TBARS), indicative of lipid peroxidation, and thiol levels, which represent antioxidant capacity [16,17]. Subsequent investigations have further revealed that the mechanisms underlying associations between mtDNA haplogroups and disease involve multiple aspects of mitochondrial biology, including bioenergetics, mitochondrial dynamics, autophagy, and metabolic reprogramming, highlighting the central role of mitochondrial homeostasis in disease pathogenesis [18,19].

East Asia has experienced rapid economic growth over recent decades, accompanied by substantial lifestyle and nutritional changes. These transitions have contributed to a marked increase in obesity and related chronic diseases. In particular, metabolic syndrome has become increasingly prevalent in the general population [20,21]. Increasing evidence suggests that mitochondrial dysfunction and mtDNA abnormalities are also closely related to metabolic syndrome and its associated metabolic disturbances [13,22,23]. Although advances in next-generation and high-throughput sequencing have greatly facilitated the discovery and characterization of mitochondrial genetic variants and their associations with complex diseases, comprehensive mitochondrial genome sequencing is not required for the targeted classification of established mtDNA haplogroups. In the present study, we therefore adopted a targeted and cost-effective genotyping strategy based on previously characterized haplogroup-defining mtSNPs, using multiplex PCR followed by probe-based Luminex genotyping to classify major mtDNA haplogroups and sub-haplogroups relevant to the Taiwanese population. This approach enabled efficient screening of established mitochondrial genetic backgrounds in a large cohort. Accordingly, in the present study, we investigated the relationships among mitochondrial DNA haplogroups, systemic oxidative stress biomarkers, and metabolic syndrome in a Taiwanese population. By analyzing mtDNA haplogroup distributions together with circulating markers of oxidative and antioxidative status, including thiobarbituric acid-reactive substances (TBARS) and serum thiols, we aimed to determine whether mitochondrial genetic background influences oxidative stress burden and susceptibility to metabolic syndrome in Asian populations.

## 2. Materials and Methods

### 2.1. Subjects

A total of 2486 subjects were recruited from our medical center in Taiwan after receiving a general health examination. Participants were not selectively excluded on the basis of common chronic diseases or medication use because the purpose of this cross-sectional study was to investigate the relationships among mtDNA haplogroups, metabolic syndrome, and systemic oxidative/antioxidative status in a general health-examination population. Furthermore, detailed information regarding specific comorbidities, antioxidant supplementation, and other medications that may influence oxidative stress biomarkers was not systematically incorporated into the present analysis. Among the subjects, 1376 were male and 1110 were female, with a mean age of 66.6 ± 14.4 years (range 20–92 years). All participants in the study provided written informed consent, while the study protocols were approved by the institutional review board at Kaohsiung Chang Gung Memorial Hospital (IRB number: 94-1092B, approved at 16 March 2006; IRB number: 201702300B0, approved at 1 February 2018). The study was performed in accordance with the Declaration of Helsinki and its text revisions. After overnight fasting, we collected venous blood samples. The PUREGENE^®^ DNA Purification kit (Gentra Systems, Minneapolis, MN, USA) was used to isolate DNA from leucocytes. Metabolic syndrome was diagnosed according to the definition issued by the National Cholesterol Education Program Adult Treatment Panel III [24]. Participants with three or more of the following components were defined as having metabolic syndrome: (1) waist circumference ≧ 102 cm in males and ≧88 cm in females; (2) triglyceride level ≧ 150 mg/dL (1.69 mmol/L); (3) high-density lipoprotein (HDL) cholesterol level ≦ 40 mg/dL (1.03 mmol/L) in males and ≦50 mg/dL (1.29 mmol/L) in females; (4) blood pressure ≧ 130/85 mm Hg or taking antihypertension medication; and (5) fasting plasma glucose level ≧ 110 mg/dL (6.1 mmol/L) or taking hypoglycemic medication. This definition included subjects with impaired fasting glucose (IFG) levels between 110 and 125 mg/dL (6.1–6.9 mmol/L), and overt diabetes with a plasma glucose level ≧ 126 mg/dL (7 mmol/L). The definition of dyslipidemia in patients with type 2 diabetes mellitus (DM) was used only when triglyceride and HDL cholesterol were at abnormal levels after control of blood glucose for more than 3 months and before the use of lipid-lowering agents.

### 2.2. Antioxidant and Oxidant Marker Assessment

Blood samples were collected into serum-separation tubes, allowed to clot at room temperature for 30 min, and centrifuged at 3000× *g* for 10 min at 4 °C. Serum was aliquoted into polypropylene tubes and stored at −80 °C until analysis. Samples were thawed once, gently mixed, and analyzed under identical conditions. Serum free thiols were measured using a colorimetric method based on Ellman and Lysko [25]. Serum was diluted fourfold with 0.1 M Tris buffer (pH 8.2), and 90 μL was transferred to a 96-well microplate. After addition of 1.9 mM 5,5′-dithiobis(2-nitrobenzoic acid (DTNB; Sigma-Aldrich, St. Louis, MO, USA) in 0.1 M phosphate buffer (pH 7.0), samples were incubated for 20 min at room temperature. Absorbance was measured at 412 nm using a Varioskan microplate reader (Thermo Scientific, Breda, The Netherlands), with 630 nm as the reference wavelength. Concentrations were calculated using the TNB molar extinction coefficient (13,600 M^−1^ cm^−1^) and expressed as μmol/L. Reagent blanks were included, and each sample was analyzed in triplicate. The intra-assay coefficient of variation (CV) was 1.9%. Serum TBARS, an index of lipid peroxidation, were determined according to Ohkawa et al. [26], with modifications. Briefly, 50 μL serum was mixed with 20 μL [SDS concentration], 150 μL 20% acetic acid, and 50 μL 0.8% thiobarbituric acid (TBA) solution (final volume, 270 μL) and heated at 95 °C for 60 min. After cooling, the reaction product was extracted with n-butanol/pyridine (15:1, *v*/*v*) and centrifuged at 5000 rpm for 5 min. Absorbance was measured at 532 nm using a DU-640 spectrophotometer (Beckman Coulter Inc., Fullerton, CA, USA, USA). A calibration curve was generated using 1,1,3,3-tetraethoxypropane (TEPP; Sigma-Aldrich, St. Louis, MO, USA), hydrolyzed to produce an MDA-equivalent standard. TBARS concentrations were calculated from the standard curve and expressed as μmol/L. Samples were analyzed in triplicate, with reagent blanks included in each assay. The intra-assay CV was 8.3%. All other reagents were of analytical grade or higher. Serum free thiol and TBARS concentrations were expressed in μmol/L.

### 2.3. Determination of Mitochondrial Haplogroup

Genomic DNA was extracted from whole blood using the PUREGENE^®^ DNA Purification Kit (Gentra Systems, Minneapolis, MN, USA). Mitochondrial haplogroups were determined using a targeted multiplex polymerase chain reaction (PCR) and probe-based Luminex genotyping approach rather than whole-mitochondrial-genome sequencing. Briefly, 24 primer pairs were used for multiplex PCR amplification of selected mtDNA regions, followed by hybridization with 94 haplogroup-specific oligonucleotide probes. The probes were covalently coupled to carboxylated fluorescent microbeads, and the amplified products were detected following hybridization and labeling with streptavidin-phycoerythrin (SA-PE). Fluorescence signals were measured using a Luminex 100 flow cytometer (Luminex Corporation, Austin, TX, USA) [27]. Based on the human mtSNP database in MITOMAP and previously established phylogenetic trees for Chinese and Japanese populations, 40 mtSNPs were selected to define 15 major mtDNA haplogroups (A, B, C, D, E, F, G, M7, M8, M9, M10, M11, N9, Y, and Z) and their constitutive sub-haplogroups (B4, B5, D4, D5, F1, F2, F3, F4, M7a, M7b, M7c, M8a, and N9a) [28,29]. The mtSNPs used for haplogroup classification have been described previously [10].

### 2.4. Statistical Analysis

All statistical analyses were performed using SPSS software (version 11.5; SPSS Inc., Chicago, IL, USA). Continuous variables are expressed as mean ± standard deviation (SD). The normality of data distribution was assessed prior to analysis, and variables with non-normal distributions were logarithmically transformed. Differences between two groups were evaluated using Student’s *t*-test, while comparisons among multiple groups were performed using one-way analysis of variance (ANOVA) followed by the least significant difference (LSD) post hoc test. Categorical variables were analyzed using the chi-square test, as appropriate. Multivariate logistic regression analyses were conducted to estimate odds ratios (ORs) and 95% confidence intervals (CIs) for the associations between mtDNA haplogroups and the five defining components of metabolic syndrome, as well as metabolic syndrome itself, with adjustment for age and sex. Bonferroni’s correction was used to correct for multiple comparisons of mtDNA haplogroups. Trend analyses were performed using linear regression with the number of MetS components entered as an ordinal variable. Unless otherwise specified, a two-sided *p* value < 0.05 was considered statistically significant. For analyses involving comparisons among the nine mtDNA haplogroups, Bonferroni correction was applied, and a *p* value < 0.0056 (0.05/9) was considered statistically significant.

## 3. Results

### 3.1. Identification and Distribution of mtDNA Haplogroups Among Taiwanese Paticipants

mtDNA haplogroups were successfully determined for 95.7% (2378/2486) of study subjects using the Luminex assay and reference data. The remaining 4.3% (108/2486) of subjects were determined as either macro-N or -M, although no further subgroup analysis was performed due to the rarity among our study cohort. The specific mtDNA nucleotide transitions used to define the macro-N and M haplogroups included the T-to-C variant at the 10,400 nucleotide position (np) as well as the G-to-C variant at the 10,398 np. In terms of specific distributions, 51% (1268/2486) of the population were in the macro-N haplogroup, while 49% (1218/2486) were in the macro-M haplogroup. Further sub-haplogroup analysis revealed B (547/2486 = 22.0%) as the most prevalent haplogroup, followed by F (474/2486 = 19.1%), D (450/2486 = 18.1%), and M7 (310/2486 = 12.5%). Other sub-haplogroups were M8 (113/2486 = 4.6%), A (112/2486 = 4.5%), N9 (79/2486 = 3.2%), G (69/2486 = 2.8%), C (61/2486 = 2.5%), E (49/2486 = 2.0%), M9 (44/2486 = 1.8%), M10 (33/2486 = 1.3%), Z (27/2486 = 1.1%), M11 (6/2486 = 0.2%), and Y (4/2486 = 0.2%).

### 3.2. Associations Between mtDNA Haplogroups and Metabolic Syndrome and Its Defining Components

To avoid introducing bias due to insufficient subject numbers among several haplogroups, we limited our analysis to haplogroups compromising more than 2% of the total study population. Consequently, nine groups including haplogroups A, B, C, D, F, G, M7, M8, N9 with a total number of 2215 subjects were selected for further study. The prevalence rates of the five defining components for metabolic syndrome in our study subjects were 54.5% (1206/2215), 35.2% (780/2215), 48.3% (1069/2215), 32.3% (716/2215), and 38.1% (844/2215) for hypertension, diabetes, obesity, high triglyceride, and low high-density lipoprotein cholesterol, respectively. To investigate the associations between these components and mtDNA haplogroups, we conducted association studies. Since we examined nine haplogroups (A, B, C, D, F, G, M7, M8, N9), we divided 0.05 by 9 to arrive at 0.0056 under Bonferroni’s correction. Thus, a *p* value of <0.0056 was considered statistically significant. A significant association between haplogroup B and diabetes was noted after multivariate analysis with correction for age and sex (OD, 1.33, *p* = 0.0055). However, despite several borderline associations, no significant associations between the five components and subjects carrying other specific mtDNA haplogroups were identified after Bonferroni’s correction (all *p* > 0.0056), as shown in Table 1. After stratifying the components, we found that 20.2% of the study population (448/2215) had one of the five components, 23.8% (527/2215) had two, 20.0% (442/2215) had three, 12.6% (279/2215) had four, and 6.1% (134/2215) had all five components. The overall prevalence rate for metabolic syndrome among our study population, defined as subjects having three or more components, was 38.6% (855/2215). The highest prevalence rate of metabolic syndrome was found in subjects carrying haplogroup B (43.9%), followed by haplogroups M8 (41.6%) and D (38.2%); meanwhile, the lowest prevalence rate was in subjects carrying haplogroup G (27.5%). We identified a significant association between the presence of metabolic syndrome and subjects carrying mtDNA haplogroup B (OD, 1.34, *p* = 0.004); however, no significant associations with metabolic syndrome were noted in subjects carrying other mtDNA haplogroups.

### 3.3. The Oxidative/Antioxidative Burden of Various mtDNA Haplogroups Stratified by Components of Metabolic Syndrome

The mean TBARS and thiol levels for overall subjects and for each studied mtDNA haplogroup are listed in Table 2. Generally, higher oxidative TBARS levels were noted in haplogroup N9, followed by haplogroups B and F. Similarly, lower antioxidative thiol levels were noted in sub-haplogroup N9, followed by B and F (Table 3). On the contrary, lower oxidative TBARS levels were noted in haplogroup G, followed by A and M8, while higher antioxidative thiol levels were noted in haplogroup C, followed by G and A. We then separated subjects into groups presenting with or without each of the five defining components for metabolic syndrome. The mean TBARS and thiol levels for the overall group and for each studied mtDNA haplogroup, stratified by presentation with or without the five defining components of metabolic syndrome, are listed in Table 2 and Table 3. In the overall group, higher TBARS and lower thiol levels were found in subjects presenting with each defining component of metabolic syndrome, including significantly higher TBARS levels in subjects with hypertension, high triglyceride and low high-density lipoprotein cholesterol. Additionally, significantly lower thiol levels were found in subjects with hypertension, diabetes, and low high-density lipoprotein cholesterol. Among the various mtDNA haplogroups, higher TBARS levels and lower thiol levels were found in most subject groups presenting with metabolic syndrome components, though these findings were not consistent across all groups.

### 3.4. The Causal Relationship Between Specific mtDNA Haplogroups, the Oxidative/Antioxidative Burden, and the Risk of Metabolic Syndrome

To investigate the causal relationship between the oxidative/antioxidative burden and the development of metabolic syndrome, and to verify whether this burden can be applied as a predictive marker for metabolic syndrome, we first calculated the mean TBARS and thiol levels of the overall subjects presenting with or without metabolic syndrome. The findings showed significantly higher TBARS levels (1.47 ± 0.84 μmol/L vs. 1.37 ± 0.78 μmol/L, *p* < 0.001) and lower thiol levels (1.79 ± 0.49 μmol/L vs. 1.94 ± 0.46 μmol/L, *p* < 0.001) in groups presenting with metabolic syndrome compared to those without (Table 4 and Table 5). We subsequently calculated the mean TBARS and thiol levels of stratified groups of overall subjects presenting with the various components (ranging from 0 to 5 components) of metabolic syndrome (Table 4 and Table 5). We identified a significant trend between the stratified subject groups presenting with more components of metabolic syndrome and elevated TBARS levels (*p* for trend < 0.001) as well as reduced thiol levels (*p* for trend < 0.001).

**Figure 1:**
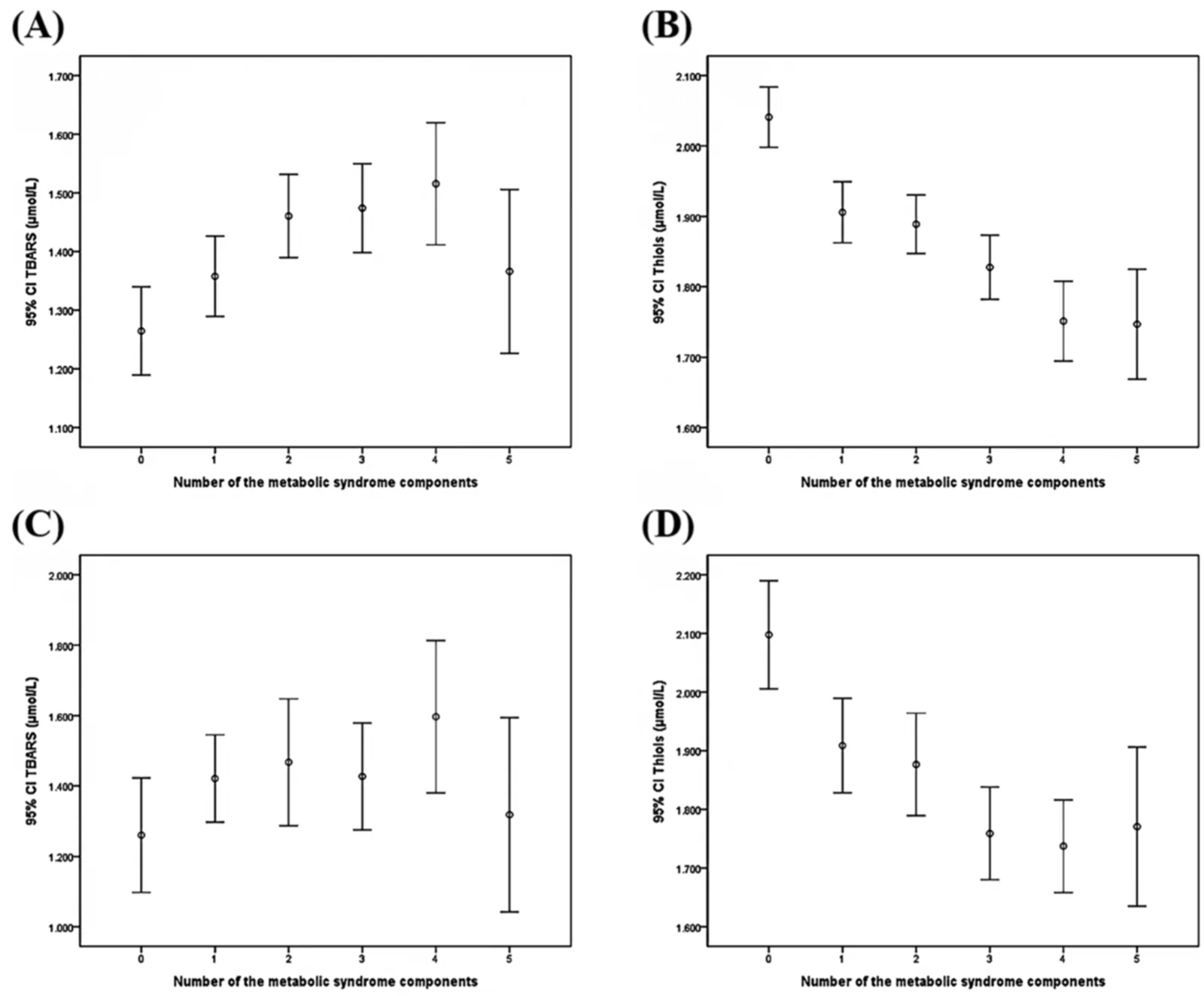
Associations between the number of metabolic syndrome components and oxidative stress markers. Mean plasma concentrations of thiobarbituric acid-reactive substances (TBARS) (**A**,**C**) and thiols (**B**,**D**) are shown according to the number of metabolic syndrome (MetS) components (0–5). Data are presented as means with 95% confidence intervals (CIs). Panels (**A**,**B**) represent the overall study population, whereas panels (**C**,**D**) represent participants carrying mitochondrial DNA (mtDNA) haplogroup B.

To further explore the causal effect of subjects carrying different mtDNA haplogroups, we calculated the mean TBARS and thiol levels in subject groups presenting with or without metabolic syndrome among the nine mtDNA haplogroups. In general, among each mtDNA haplogroup, subjects presenting with metabolic syndrome exhibited higher TBARS and lower thiol levels than those without (Table 4 and Table 5). However, only some mtDNA haplogroups achieved statistically significant lower thiol levels. We then calculated the mean TBARS and thiol levels among the stratified groups of subjects with various components of metabolic syndrome (ranging from 0 to 5 components) among the nine mtDNA haplogroups (Table 4 and Table 5). Significant correlations were observed between more metabolic syndrome components and decreasing thiol levels among individuals with mtDNA haplogroups B (*p* for trend < 0.001), F (*p* for trend = 0.007), and M8 (*p* for trend = 0.028). In contrast, only borderline associations were observed between higher TBARS levels and mtDNA haplogroups A (*p* for trend = 0.044) and F (*p* for trend = 0.039) (Figure 1).

## 4. Discussion

Mitochondrial genetic variation has increasingly been recognized as an important contributor to differences in mitochondrial function and susceptibility to complex diseases. Advances in high-throughput and next-generation sequencing technologies have substantially expanded our ability to identify and characterize mtDNA variants and to define mitochondrial haplogroup structures at the population level. However, the present study was not designed to discover novel mtDNA variants by sequencing. Instead, we used a targeted genotyping approach based on previously established haplogroup-defining mtSNPs, employing multiplex PCR and probe-based Luminex detection. This strategy enabled efficient classification of established mtDNA haplogroups in a large study population and provided a practical approach for evaluating their associations with systemic oxidative stress and medical conditions. Although comprehensive mitochondrial genome sequencing can provide detailed information on mtDNA variation, targeted genotyping of established haplogroup-defining variants is appropriate when the primary objective is the classification of known mitochondrial genetic backgrounds. In the present study, we characterized the distribution of mtDNA haplogroups in a large Taiwanese cohort and investigated their associations with oxidative stress biomarkers and metabolic syndrome. Our findings indicate that the Taiwanese population is predominantly composed of macrohaplogroups N (51%) and M (49%), with haplogroups B, F, D, and M7 representing the most common lineages. Importantly, we identified a significant association between haplogroup B and both diabetes and metabolic syndrome, accompanied by a distinct oxidative profile characterized by higher TBARS levels and lower thiol concentrations.

The haplogroup distribution observed in our cohort is consistent with patterns reported in other East Asian populations [29,30,31,32,33,34] (Table 6). Studies from mainland China have shown that haplogroups M, D, B, and F collectively represent the majority of mitochondrial lineages [28,29]. Similar distributions have been observed in regional datasets from southern China, but differ slightly from those of the central region of China [9]. In populations such as Japan, Korea and northern China, haplogroup D is particularly prevalent, whereas populations in Southeast Asia, including the Philippines and Malaysia, demonstrate greater representations of haplogroups B and M, along with additional diversity involving haplogroups E and F [35]. Overall, these findings indicate that the mitochondrial genetic background of the Taiwanese population reflects the broader East Asian genetic landscape, while retaining regional characteristics shaped by historical migration and demographic processes. Recent population and mitochondrial genetic studies have further emphasized that mtDNA haplogroup distributions reflect complex demographic histories and should be interpreted in the context of geographic and population-specific genetic backgrounds.

Mitochondrial genetic variation has long been implicated in the regulation of cellular oxidative stress [5,36]. Variants within the coding and control regions of mtDNA may influence mitochondrial respiratory-chain function, electron transport efficiency, and mitochondrial bioenergetics, thereby affecting reactive oxygen species (ROS) production and redox balance [2,36]. Because mtDNA encodes essential components of the oxidative phosphorylation system, differences in mitochondrial genetic background may alter the efficiency of electron transport and potentially increase electron leakage under conditions of increased metabolic demand or metabolic stress. However, mitochondrial redox homeostasis is determined not only by ROS generation but also by mitochondrial biogenesis, mtDNA maintenance, mitochondrial quality control, and the capacity of cellular antioxidant systems [8,13,22,23]. Thus, mtDNA haplogroups may influence systemic oxidative status through multiple interconnected pathways rather than through a single genetic variant or mechanism [37]. In the present study, carriers of different mtDNA haplogroups exhibited heterogeneous oxidative stress profiles, suggesting that mitochondrial genetic background may modify individual susceptibility to oxidative imbalance.

Among the haplogroups examined, haplogroup B demonstrated the most notable pro-oxidative profile, with relatively higher TBARS measurements than other haplogroups. One possible explanation involves characteristic mtDNA variants defining this haplogroup. Haplogroup B contains a 9-base-pair deletion in the intergenic region between the cytochrome c oxidase II gene and the mitochondrial TK2 thymidine kinase gene [38]. Although this deletion itself is considered nonfunctional, haplogroup B carriers frequently harbor the T-to-C transition at nucleotide position 16,189, which forms a polycytosine tract in the control region [39]. Previous studies have suggested that this variant may affect mtDNA replication efficiency and may be associated with reduced mtDNA copy number and diminished mitochondrial biogenesis [40]. Such alterations could potentially compromise mitochondrial adaptation to metabolic stress and affect oxidative phosphorylation efficiency, thereby increasing susceptibility to ROS accumulation. Increased mitochondrial ROS may subsequently promote lipid peroxidation, which could contribute to the higher TBARS levels observed in haplogroup B carriers. At the same time, sustained oxidative stress may increase the consumption or oxidative modification of thiol-containing molecules, potentially contributing to a reduction in circulating thiol concentrations. Therefore, the oxidative profile observed in haplogroup B may reflect the combined effects of altered mitochondrial homeostasis and systemic redox imbalance. Nevertheless, because mtDNA copy number, mitochondrial respiration, and ROS production were not directly measured in the present study, this proposed mechanism remains hypothetical and requires further functional investigation.

The observed combination of higher TBARS and lower thiol concentrations in metabolically affected individuals may reflect different but interconnected consequences of oxidative imbalance. Increased mitochondrial ROS can initiate lipid peroxidation, resulting in the formation of reactive lipid-derived products that contribute to TBARS measurements. Conversely, sustained oxidative stress can consume or oxidatively modify thiol-containing molecules and thereby reduce the circulating thiol pool. Thus, the simultaneous elevation of TBARS and reduction in thiols may indicate a shift toward a more pro-oxidative systemic environment. Nevertheless, TBARS and serum free thiols represent indirect and complementary measures of systemic redox status and should not be interpreted as direct measurements of mitochondrial ROS generation or the activity of specific antioxidant enzymes.

The contrasting thiols profiles observed across haplogroups provide an additional perspective on the relationship between mitochondrial genetic background and systemic redox homeostasis. In particular, haplogroup D carriers exhibited the highest circulating thiol levels, which may indicate relatively greater preservation of systemic redox buffering capacity. Thiol-containing molecules, including glutathione and cysteine, represent important components of antioxidant defense, while circulating free thiols contribute to the maintenance of extracellular redox balance and are influenced by the dynamic equilibrium between oxidative consumption and antioxidant regeneration. Thus, the higher thiol concentrations observed in haplogroup D may reflect a comparatively greater capacity to maintain systemic redox homeostasis rather than a direct increase in mitochondrial antioxidant activity. Previous studies in Japanese populations have reported an association between the D5 sub-haplogroup and longevity, suggesting that certain mitochondrial genetic backgrounds may be associated with greater physiological resilience [41]. Although this observation is consistent with the relatively preserved thiol status observed in haplogroup D carriers in our study, it did not establish a direct relationship between haplogroup D, longevity, and antioxidant capacity. Taken together, the findings suggest that different mtDNA haplogroups may be associated with distinct systemic redox phenotypes, potentially reflecting differences in mitochondrial adaptation to metabolic stress. However, these associations should be interpreted cautiously because circulating TBARS and thiol concentrations are indirect measures of systemic redox status and cannot by themselves establish specific mitochondrial mechanisms.

Our findings provide evidence linking oxidative stress to the development and progression of metabolic syndrome. Individuals with metabolic syndrome exhibited elevated TBARS levels and reduced thiol concentrations, reflecting enhanced lipid peroxidation and compromised antioxidant defenses [16,42]. Moreover, when subjects were stratified according to the number of metabolic syndrome components, we observed a progressive increase in TBARS levels and a concomitant decrease in thiol concentrations as the number of metabolic abnormalities increased. These findings indicate that worsening metabolic dysfunction is accompanied by a gradual shift toward a pro-oxidative state. Oxidative stress has been shown to disrupt insulin signaling, promote chronic inflammation, and contribute to endothelial dysfunction, all of which are key mechanisms underlying cardiometabolic disease [42,43]. These findings are consistent with our observation that metabolic abnormalities are associated with systemic oxidative imbalance. Interestingly, although both TBARS and thiol levels were associated with metabolic abnormalities, the relationship between thiol levels and metabolic syndrome appeared more consistent across mtDNA haplogroups. In particular, decreasing thiol levels were significantly associated with increasing numbers of metabolic syndrome components in several haplogroups, including haplogroup B. In contrast, trends involving TBARS levels were less consistent. These findings suggest that serum thiol concentrations may represent a more sensitive indicator of systemic redox imbalance in metabolic syndrome.

A key finding of this study is the interaction between mitochondrial genetic background, oxidative stress, and metabolic disease susceptibility. Haplogroup B was associated with both increased oxidative stress and a higher prevalence of metabolic syndrome. Recent systemic evidence further supports associations between mtDNA variants, copy number, haplogroups, and type 2 diabetes across different ethnic populations [44]. Given the central role of mitochondria in energy metabolism, mtDNA variation may influence disease risk through effects on bioenergetic efficiency and reactive oxygen species production. The potential mechanism may involve a sequence of interconnected events in which mtDNA-associated differences in mitochondrial function alter respiratory-chain efficiency and ROS generation, while metabolic stress further increases mitochondrial demand and oxidative burden. Persistent oxidative stress may then promote lipid peroxidation, impair redox-buffering capacity, interfere with insulin signaling, and activate inflammatory pathways, thereby contributing to metabolic dysfunction [23]. In this context, mitochondrial genetic background may not directly cause metabolic syndrome but may modify individual susceptibility to metabolic stress. Under conditions of metabolic stress, individuals carrying specific mitochondrial haplogroups may be more vulnerable to oxidative damage [45], which may contribute to the development of metabolic abnormalities over time, including metabolic syndrome. This interpretation is also compatible with emerging evidence that mitochondrial phenotypes are influenced by interactions between mtDNA and nuclear genetic backgrounds, as well as by environmental and metabolic conditions [8,37]. Thus, the effect of a particular mtDNA haplogroup may not be uniform across individuals or metabolic environments. The present findings are particularly relevant in light of the rapidly increasing prevalence of metabolic syndrome in East and Southeast Asia. Epidemiological studies have reported substantial increases in metabolic syndrome prevalence across the region over recent decades, largely driven by rapid urbanization, aging populations, and lifestyle changes [20,21]. Given the widespread distribution of haplogroups such as B, D, and F in Asian populations, interactions between mitochondrial genetic background and environmental factors may play an important role in shaping population susceptibility to metabolic diseases.

Taken together, our findings support a potential model in which mitochondrial genetic background modifies susceptibility to systemic oxidative imbalance under metabolic stress. In this model, mtDNA haplogroup-associated differences in mitochondrial bioenergetics, respiratory-chain function, or mtDNA maintenance may influence ROS production and mitochondrial adaptability [23]. Increased oxidative burden may subsequently promote lipid peroxidation and depletion or modification of thiol-based redox defenses, as reflected by higher TBARS and lower thiol concentrations. These redox disturbances may interact with insulin resistance, inflammation, dyslipidemia, and other components of metabolic syndrome, potentially creating a reciprocal cycle between mitochondrial dysfunction and metabolic stress. Nevertheless, this model remains mechanistic and hypothetical because the present study did not directly measure mitochondrial respiration, ROS generation, mtDNA copy number, mitochondrial biogenesis, or antioxidant enzyme activity.

Several limitations should be considered when interpreting our findings. First, the cross-sectional design of this study limits the ability to establish causal relationships between mtDNA variation, oxidative stress, and metabolic syndrome. Second, oxidative stress was evaluated using a limited number of biomarkers. Although TBARS and serum free thiols provide complementary information regarding lipid peroxidation and systemic antioxidant/redox-buffering capacity, respectively, they do not encompass the full spectrum of oxidative stress and antioxidant defense pathways. Future investigations incorporating additional parameters related to reactive oxygen species generation and antioxidant defense, such as SOD, CAT, glutathione, hydrogen peroxide, superoxide-related indices, and nitrite/nitric oxide-related markers, may provide a more comprehensive characterization of systemic redox status and further strengthen the mechanistic interpretation of the present findings. Finally, lifestyle and environmental factors that influence oxidative stress, such as diet, physical activity, smoking, and other comorbid diseases, were not fully accounted for in this analysis.

## 5. Conclusions

In conclusion, our study demonstrates that mtDNA haplogroups are associated with differences in systemic oxidative stress and may influence susceptibility to metabolic syndrome in several Asian populations. Haplogroup B was particularly associated with increased oxidative stress and a higher prevalence of metabolic syndrome. These findings highlight the potential contribution of mitochondrial genetic background to metabolic disease risk and underscore the importance of considering mitochondrial genetics in future studies of metabolic disorders. In clinical practice, our observation of antioxidant thiol concentrations as a sensitive indicator of systemic redox imbalance may be employed as a valuable diagnostic biomarker for metabolic syndrome.

## Figures and Tables

**Table 1:** Multivariate logistic regression analysis of associations between mtDNA haplogroups and metabolic syndrome and its components, adjusted for age and sex.

mtDNA Haplogroup	Overall	Hypertension		Diabetes mellitus		Obesity		Hypertriglyceridemia		Reduced HDL-C		Metabolic Syndrome	
	(*n* = 2215)	(*n* = 1206)		(*n* = 780)		(*n* = 1069)		(*n* = 716)		(*n* = 844)		(*n* = 855)	
	% (n)	OR (95% CI)	*p*	OR (95% CI)	*p*	OR (95% CI)	*p*	OR (95% CI)	*p*	OR (95% CI)	*p*	OR (95% CI)	*p*
A	4.5 (112)	1.00 (0.66–1.54)	0.99	0.65 (0.42–1.00)	0.05	0.88 (0.60–1.29)	0.52	0.90 (0.60–1.36)	0.63	0.95 (0.64–1.42)	0.8	0.64 (0.42–0.98)	0.04
B	22.0 (547)	1.27 (1.02–1.53)	0.03	1.33 (1.09–1.63)	<0.01	1.10 (0.91–1.34)	0.34	0.93 (0.76–1.15)	0.5	1.17 (0.96–1.42)	0.13	1.34 (1.10–1.64)	<0.01
C	2.5 (61)	1.20 (0.67–2.17)	0.55	1.22 (0.72–2.09)	0.46	1.03 (0.62–1.71)	0.92	1.01 (0.59–1.74)	0.96	0.82 (0.48–1.42)	0.49	0.84 (0.49–1.45)	0.54
D	18.1 (450)	1.14 (0.90–1.45)	0.27	0.85 (0.68–1.07)	0.17	1.13 (0.92–1.39)	0.25	1.02 (0.82–1.27)	0.86	0.93 (0.75–1.15)	0.5	1.01 (0.81–1.25)	0.93
F	19.1 (474)	0.89 (0.71–1.12)	0.33	1.00 (0.80–1.24)	0.98	0.85 (0.60–1.05)	0.13	0.89 (0.71–1.10)	0.28	1.00 (0.81–1.24)	0.99	0.92 (0.74–1.14)	0.43
G	2.8 (69)	0.81 (0.47–1.42)	0.47	0.61 (0.34–1.09)	0.09	0.72 (0.44–1.17)	0.18	0.76 (0.44–1.30)	0.31	0.65 (0.38–1.11)	0.12	0.65 (0.38–1.13)	0.12
M7	12.5 (310)	0.76 (0.58–0.99)	0.04	0.99 (0.76–1.28)	0.92	0.86 (0.67–1.09)	0.2	1.13 (0.88–1.46)	0.33	1.05 (0.82–1.35)	0.7	0.95 (0.74–1.23)	0.71
M8	4.6 (113)	1.09 (0.71–1.68)	0.69	1.18 (0.79–1.75)	0.42	1.44 (0.98–2.11)	0.06	1.26 (0.85–1.87)	0.25	0.97 (0.65–1.44)	0.88	1.13 (0.76–1.66)	0.56
N9	3.2 (79)	0.61 (0.37–1.01)	0.06	0.84 (0.51–1.37)	0.48	1.21 (0.77–1.90)	0.41	1.51 (0.96–2.39)	0.08	0.92 (0.58–1.48)	0.73	0.82 (0.51–1.32)	0.4

mtDNA, mitochondrial DNA; HDL-C, high-density lipoprotein cholesterol; OR, odds ratio; CI, confidence interval. Data are presented as *n* (%) for the overall distribution of mtDNA haplogroups. ORs and 95% CIs were obtained from multivariate logistic regression models adjusted for age and sex. A two-sided *p* < 0.05 was considered statistically significant. Bonferroni correction was applied for comparisons among the nine mtDNA haplogroups, with a corrected significance threshold of *p* < 0.0056 (0.05/9).

**Table 2:** Mean plasma TBARS levels (µmol/L) according to mtDNA haplogroups, stratified by components of metabolic syndrome.

mtDNA Haplogroup	Mean (SD)	Hypertension		Diabetes mellitus		Obesity		Hypertriglyceridemia		Reduced HDL-C	
		Yes	No	Yes	No	Yes	No	Yes	No	Yes	No
A	1.38 (0.73)	1.51 (0.73)	1.22 (0.71) *	1.33 (0.59)	1.40 (0.78)	1.35 (0.78)	1.40 (0.69)	1.36 (0.76)	1.39 (0.72)	1.40 (0.61)	1.36 (0.79)
B	1.43 (0.84)	1.52 (0.91)	1.29 (0.72) *	1.43 (0.85)	1.42 (0.83)	1.46 (0.91)	1.39 (0.77)	1.50 (0.90)	1.39 (0.81)	1.40 (0.86)	1.44 (0.83)
C	1.40 (0.72)	1.37 (0.73)	1.45 (0.72)	1.42 (0.84)	1.39 (0.64)	1.54 (0.75)	1.26 (0.67)	1.58 (0.89)	1.32 (0.61)	1.46 (0.85)	1.38 (0.65)
D	1.40 (0.79)	1.48 (0.84)	1.31 (0.71) *	1.42 (0.76)	1.40 (0.80)	1.40 (0.83)	1.40 (0.75)	1.50 (0.86)	1.36 (0.75)	1.32 (0.75)	1.45 (0.81)
F	1.42 (0.85)	1.58 (0.92)	1.23 (0.73) *	1.46 (0.89)	1.40 (0.83)	1.46 (0.87)	1.39 (0.84)	1.53 (0.87)	1.37 (0.84)	1.35 (0.80)	1.46 (0.88)
G	1.30 (0.70)	1.48 (0.58)	1.17 (0.76)	1.52 (0.92)	1.24 (0.62)	1.31 (0.67)	1.30 (0.73)	1.37 (0.74)	1.28 (0.70)	1.18 (0.67)	1.35 (0.72)
M7	1.42 (0.81)	1.46 (0.84)	1.37 (0.79)	1.43 (0.85)	1.41 (0.80)	1.45 (0.77)	1.39 (0.86)	1.54 (0.82)	1.35 (0.80)	1.45 (0.79)	1.39 (0.83)
M8	1.38 (0.69)	1.39 (0.60)	1.38 (0.80)	1.35 (0.66)	1.41 (0.72)	1.49 (0.73)	1.22 (0.60) *	1.44 (0.82)	1.35 (0.61)	1.44 (0.73)	1.35 (0.67)
N9	1.44 (0.70)	1.56 (0.65)	1.33 (0.74)	1.47 (0.68)	1.43 (0.72)	1.53 (0.72)	1.34 (0.68)	1.53 (0.70)	1.37 (0.71)	1.35 (0.63)	1.50 (0.75)
Overall	1.41 (0.80)	1.51 (0.85)	1.30 (0.74) **	1.43 (0.82)	1.40 (0.80)	1.44 (0.83)	1.38 (0.78)	1.50 (0.85)	1.37 (0.78) **	1.38 (0.78)	1.43 (0.82) **

TBARS: thiobarbituric acid-reactive substances; mtDNA: mitochondrial DNA; HDL-C: high-density lipoprotein cholesterol. Data are presented as mean (standard deviation, SD). *p* values represent comparisons between groups with and without each metabolic syndrome component within the same mtDNA haplogroup. * *p* < 0.05 and ** *p* < 0.01 were considered statistically significant.

**Table 3:** Mean plasma thiol levels (µmol/L) according to mtDNA haplogroups, stratified by components of metabolic syndrome.

mtDNA Haplogroup	Mean (SD)	Hypertension		Diabetes mellitus		Obesity		Hypertriglyceridemia		Reduced HDL-C	
		Yes	No	Yes	No	Yes	No	Yes	No	Yes	No
A	1.92 (0.66)	1.77 (0.46)	2.10 (0.80) *	1.71 (0.68)	2.00 (0.64) *	1.81 (0.43)	2.01 (0.79)	1.80 (0.59)	1.97 (0.68)	1.89 (0.88)	1.94 (0.49)
B	1.87 (0.44)	1.78 (0.41)	1.98 (0.46)	1.76 (0.45)	1.93 (0.43)	1.81 (0.43)	1.91 (0.45)	1.81 (0.40)	1.89 (0.46)	1.78 (0.42)	1.92 (0.45)
C	1.94 (0.51)	1.81 (0.53)	2.12 (0.43) *	1.82 (0.60)	2.02 (0.43)	1.96 (0.52)	1.93 (0.51)	1.67 (0.62)	2.08 (0.39) *	1.81 (0.67)	2.02 (0.39)
D	1.88 (0.47)	1.81 (0.50)	1.97 (0.42)	1.79 (0.51)	1.92 (0.45)	1.84 (0.48)	1.93 (0.47)	1.93 (0.50)	1.86 (0.46)	1.87 (0.54)	1.89 (0.43)
F	1.87 (0.44)	1.80 (0.43)	1.95 (0.43)	1.77 (0.47)	1.92 (0.41)	1.84 (0.46)	1.89 (0.41)	1.85 (0.47)	1.87 (0.42)	1.83 (0.50)	1.89 (0.39)
G	1.93 (0.52)	1.68 (0.45)	2.12 (0.50) **	1.97 (0.59)	1.91 (0.51)	1.90 (0.35)	1.95 (0.61)	2.01 (0.54)	1.90 (0.52)	1.95 (0.55)	1.92 (0.52)
M7	1.91 (0.54)	1.85 (0.49)	1.98 (0.58) *	1.86 (0.54)	1.94 (0.54)	1.87 (0.58)	1.95 (0.50)	1.88 (0.61)	1.93 (0.50)	1.87 (0.60)	1.94 (0.50)
M8	1.87 (0.45)	1.72 (0.42)	2.05 (0.42)	1.75 (0.48)	1.94 (0.41)	1.83 (0.46)	1.92 (0.44)	1.78 (0.41)	1.92 (0.47)	1.77 (0.40)	1.93 (0.47)
N9	1.82 (0.40)	1.79 (0.37)	1.86 (0.43)	1.78 (0.44)	1.84 (0.39)	1.86 (0.40)	1.78 (0.41)	1.87 (0.44)	1.79 (0.38)	1.76 (0.32)	1.87 (0.44)
Overall	1.88 (0.48)	1.79 (0.45)	1.98 (0.49) **	1.79 (0.50)	1.93 (0.46) **	1.84 (0.46)	1.92 (0.49)	1.85 (0.49)	1.89 (0.47)	1.83 (0.52)	1.91 (0.45) *

mtDNA: mitochondrial DNA; HDL-C: high-density lipoprotein cholesterol. Data are presented as mean (standard deviation, SD). *p* values represent comparisons between groups with and without each metabolic syndrome component within the same mtDNA haplogroup. * *p* < 0.05 and ** *p* < 0.01 were considered statistically significant.

**Table 4:** Mean plasma TBARS levels (µmol/L) according to mtDNA haplogroups and number of metabolic syndrome components.

mtDNA Haplogroup	Metabolic Syndrome		*p-*Value	0 Component	1 Component	2 Components	3 Components	4 Components	5 Components	*p* for Trend
	Yes	No								
A	1.39 (0.66)	1.38 (0.76)	0.952	1.13 (0.72)	1.25 (0.66)	1.62 (0.81)	1.64 (0.71)	1.27 (0.53)	0.97 (0.47)	0.044 *
B	1.48 (0.92)	1.39 (0.78)	0.138	1.26 (0.76)	1.42 (0.67)	1.44 (0.89)	1.45 (0.88)	1.60 (0.98)	1.32 (0.85)	0.19
C	1.48 (0.84)	1.36 (0.64)	0.506	1.40 (0.73)	1.23 (0.47)	1.41 (0.71)	1.29 (0.67)	1.53 (0.85)	2.17 (1.72)	0.62
D	1.44 (0.80)	1.38 (0.79)	0.504	1.36 (0.68)	1.28 (0.78)	1.50 (0.86)	1.44 (0.70)	1.53 (1.00)	1.23 (0.63)	0.207
F	1.49 (0.89)	1.38 (0.83)	0.215	1.16 (0.70)	1.42 (0.86)	1.51 (0.87)	1.53 (0.90)	1.42 (0.90)	1.45 (0.84)	0.039 *
G	1.38 (0.75)	1.27 (0.69)	0.565	1.17 (0.80)	1.18 (0.44)	1.49 (0.69)	1.46 (0.76)	1.42 (0.71)	0.28 (0.00)	0.398
M7	1.49 (0.85)	1.37 (0.79)	0.234	1.34 (0.92)	1.34 (0.72)	1.42 (0.74)	1.45 (0.87)	1.50 (0.71)	1.59 (1.00)	0.809
M8	1.47 (0.71)	1.30 (0.68)	0.209	1.33 (0.59)	1.31 (0.74)	1.28 (0.69)	1.47 (0.72)	1.46 (0.73)	1.51 (0.71)	0.898
N9	1.51 (0.64)	1.40 (0.74)	0.495	1.24 (0.77)	1.53 (0.72)	1.38 (0.76)	1.37 (0.64)	1.66 (0.59)	1.63 (0.81)	0.788
Overall	1.47 (0.84)	1.37 (0.78)	0.006 **	1.27 (0.75)	1.35 (0.74)	1.47 (0.83)	1.47 (0.81)	1.52 (0.88)	1.37 (0.82)	<0.001 **

TBARS: thiobarbituric acid-reactive substances; mtDNA: mitochondrial DNA. Data are presented as mean (standard deviation, SD). *p* values compare groups with and without metabolic syndrome within each mtDNA haplogroup. *p* for trend was calculated using linear regression with the numbers of metabolic syndrome components entered as an ordinal variable. A two-sided *p* < 0.05 was considered statistically significant. * *p* < 0.05; ** *p* < 0.01.

**Table 5:** Mean plasma thiol levels (µmol/L) according to mtDNA haplogroups and number of metabolic syndrome components.

mtDNA Haplogroup	Metabolic Syndrome		*p-*Value	0 Component	1 Component	2 Components	3 Components	4 Components	5 Components	*p* for Trend
	Yes	No								
A	1.66 (0.48)	2.03 (0.69)	0.007 **	2.04 (0.40)	2.15 (0.98)	1.91 (0.50)	1.71 (0.39)	1.57 (0.58)	1.67 (0.60)	0.087
B	1.75 (0.41)	1.95 (0.45)	<0.001 **	2.10 (0.43)	1.91 (0.44)	1.87 (0.45)	1.75 (0.44)	1.74 (0.36)	1.77 (0.42)	<0.001 *
C	1.76 (0.61)	2.06 (0.40)	0.024 *	2.24 (0.22)	2.00 (0.42)	2.00 (0.45)	1.94 (0.32)	1.63 (0.78)	1.52 (0.73)	0.097
D	1.84 (0.56)	1.91 (0.41)	0.455	1.95 (0.46)	1.92 (0.39)	1.87 (0.38)	1.90 (0.59)	1.82 (0.53)	1.70 (0.48)	0.352
F	1.80 (0.48)	1.90 (0.41)	0.065	2.04 (0.36)	1.83 (0.34)	1.87 (0.47)	1.85 (0.46)	1.74 (0.49)	1.76 (0.50)	0.007 *
G	1.91 (0.53)	1.93 (0.53)	0.858	2.11 (0.51)	1.74 (0.60)	1.85 (0.44)	1.94 (0.66)	1.80 (0.25)	2.36 (0.00)	0.377
M7	1.84 (0.57)	1.96 (0.52)	0.054	2.03 (0.45)	1.93 (0.44)	1.91 (0.61)	1.87 (0.60)	1.80 (0.61)	1.82 (0.37)	0.315
M8	1.73 (0.37)	1.99 (0.48)	0.037 *	2.10 (0.37)	1.92 (0.41)	1.98 (0.59)	1.86 (0.36)	1.63 (0.32)	1.49 (0.39)	0.028 *
N9	1.81 (0.38)	1.83 (0.42)	0.602	1.94 (0.51)	1.81 (0.35)	1.80 (0.43)	1.73 (0.26)	1.86 (0.49)	1.98 (0.49)	0.386
Overall	1.79 (0.49)	1.94 (0.46)	<0.001 **	2.04 (0.43)	1.91 (0.47)	1.89 (0.48)	1.83 (0.50)	1.75 (0.48)	1.75 (0.46)	<0.001 **

mtDNA: mitochondrial DNA. Data are presented as mean (standard deviation, SD). *p* values compare groups with and without metabolic syndrome within each mtDNA haplogroup. *p* for trend was calculated using linear regression with the numbers of metabolic syndrome components entered as an ordinal variable. A two-sided *p* < 0.05 was considered statistically significant. * *p* < 0.05; ** *p* < 0.01.

**Table 6:** Frequencies (%) of mtDNA haplogroups in Taiwanese and neighboring East and Southeast Asian populations.

mtDNA Haplogroup	Japan	Korea	North China	Central China	South China	Taiwan	Philippines	Malaysia
A	5–8	8–10	6–10	4–8	1–4	3–6	<2	Rare
B	10–15	12–16	5–12	8–15	15–25	18–25	25–40	20–35
C	1–4	2–4	4–10	2–6	1–3	1–4	Rare	Rare
D	30–40	30–35	20–30	18–25	10–18	15–22	3–8	<5
E	Rare	Rare	Rare	Rare	Rare	1–3	15–30	5–15
F	5–10	8–12	5–10	8–15	15–25	15–22	10–20	15–25
G	3–6	8–12	3–8	2–5	1 = 3	1–4	Rare	Rare
M7	8–15	8–12	2–6	5–10	8–15	10–15	5–10	5–15
M8	3–5	3–7	4–8	2–5	1–3	3–6	Rare	Rare
N9	2–5	1–4	1–4	1–3	1–3	2–5	Rare	Rare
Y	1–3	<2	1–3	<2	Rare	<1	Rare	Rare
Z	<1	<2	<3	Rare	Rare	<2	Rare	Rare

Rare: frequency < 0.5%.

## Data Availability

The original contributions presented in this study are included in the article. Further inquiries can be directed to the corresponding author(s).
